# 4D nucleome: dynamic three-dimensional genome organization over time

**DOI:** 10.1038/s12276-024-01248-5

**Published:** 2024-04-25

**Authors:** Hyoung-Pyo Kim

**Affiliations:** 1 Department of Tropical Medicine, Institute of Tropical Medicine, Yonsei University College of Medicine, Seoul, Korea; 2 Brain Korea 21 PLUS Project for Medical Science, Yonsei University College of Medicine, Seoul, Korea; 3 Yonsei Genome Center, Yonsei University College of Medicine, Seoul, Korea; 4 Division of Biology, Pohang University of Science and Technology, Pohang, Korea

**Keywords:** Chromatin remodelling, Genetics research

The chromatin architecture of mammalian genomes is intricately organized into a multilayered three-dimensional structure that features compartments, topologically associating domains (TADs), and chromatin loops^1,2^. Individual chromosomes segregate into either active or inactive compartments through homotypic interactions among genomic regions with similar transcriptional and chromatin states. TADs exhibit preferential interactions within their chromatin and are delineated by distinct boundaries, frequently forming loops. Chromosomal spatial folding and organization in the nucleus crucially regulate gene expression and cellular function, impacting both normal development and the onset of various diseases^3–5^. Exploring the 3D organization of DNA within the nucleus and its temporal changes is a key focus of the 4D Nucleome Project^6^. This special issue provides comprehensive reviews of technological advances in the investigation of the 3D organization of DNA in the nucleus and the functional implications of the 4D nucleome in physiology and human disease.

Han et al.^7^ explored significant advancements in experimental techniques developed over the past decade to elucidate the multilayered organization of the 3D genome and its impact on transcription and other biological functions. They initially introduced sequencing-based approaches for mapping genome-wide chromatin interactions, exemplified by Hi–C, which utilizes proximity ligation followed by deep sequencing. This method was subsequently integrated with other measurements to reveal chromosomal topological organization across various biological parameters. They also pioneered imaging technologies for direct investigation of 3D chromatin architecture at a single-cell resolution, leveraging advancements in superresolution microscopy, and the development of high-throughput Oligopaint library methods. Additionally, they emphasized innovations in single-cell multimodal approaches, which enable the simultaneous measurement of gene expression and epigenetic states, elucidating the intricate spatiotemporal dynamics governing gene regulation.

The precise regulation of gene expression by *cis*-regulatory elements, such as promoters and enhancers, is highly complex and crucial for proper development, tissue differentiation, and response to environmental cues or diseases. Understanding these regulatory mechanisms is essential for unraveling the complexities of cellular processes and disease pathology. Friedman et al.^8^ investigated recent progress in the identification of enhancer–promoter interactions within the framework of 3D genome architecture. Additionally, they reviewed the core characteristics of the molecular mechanisms governing the communication of enhancers with their respective target genes. Moreover, they examined how dysregulation of enhancer–promoter interactions can lead to transcriptional changes associated with disease, highlighting its potential as a promising target for therapeutic interventions.

Yoon et al.^9^ explored recent advancements in understanding the complexities of 3D chromatin structure and its significance in regulating gene expression, particularly in the context of cancer. Specifically, their discussion explored the impact of dysregulated 3D chromatin structure on transcriptional regulation, which can activate oncogenes or deactivate tumor suppressor genes. This dysregulation arises from structural variations such as deletions, inversions, translocations, and chromothripsis, resulting in significant alterations in the chromatin landscape. Their discussion extended to the relationship of 3D chromatin architecture with extrachromosomal DNA, which is considered a major factor in tumor heterogeneity and contributes to drug resistance and poor prognosis.

Viral infections instigate alterations in diverse biological processes within host cells, exerting influence on gene expression and replication, latency, and oncogenic transformation. Kim et al.^10^ highlighted the importance of understanding how viral infections reshape the 3D chromatin structure of both viral and host genomes and how this reshaping impacts viral and host gene regulation. They explored the intricate interactions between viral and host genomes at various levels, encompassing episomal tethering and genome integrations. These interactions give rise to novel local chromatin environments, exerting influence on diverse viral and host genes. They further explored the roles played by cellular chromatin-organizing factors and viral-encoded proteins in modulating diverse host-viral interactions. These factors significantly influence viral processes and the dynamic organization of the cellular 4D nucleome.

Phase separation is a process in which a single homogeneous mixture segregates into two distinct phases. It is currently understood to be the basis for the formation of non-membrane-bound compartments and the creation of distinct microenvironments that facilitate the compartmentalization of specific biochemical reactions. Park et al.^11^ presented two potential working models, self-association-induced phase separation and bridging-induced phase separation, to elucidate chromosomal phase separation and its involvement in chromosome function. The authors also provided physiologically relevant examples of these two mechanisms and proposed that these mechanisms may be commonly applicable to other chromosomal phase-separated condensates.

Recent advances in microscopy techniques have revealed an intriguing concept termed ‘transcriptional condensates,’ indicating the pivotal role of phase separation in gene expression control. Ryu et al.^12^ highlighted current advances in elucidating the composition and characteristics of transcriptional condensates, particularly within the context of chromatin dynamics. They also explored pioneering techniques enabling the manipulation of these condensates, demonstrating their responsiveness to cellular signals and their correlation with transcriptional bursting events occurring within the eukaryotic cell nucleus.

These articles collectively underscore the potential for studies to move beyond a simplistic linear representation of the genome to explore its dynamically organized three-dimensional structure within living cells. Furthermore, leveraging comprehensive 4D nucleome data in future analyses may offer invaluable insights into disease mechanisms and discover new therapeutic possibilities for combating various conditions.
